# Differentiating Cannabis Products: Drugs, Food, and Supplements

**DOI:** 10.3389/fphar.2022.906038

**Published:** 2022-06-27

**Authors:** Arash Salehi, Keely Puchalski, Yalda Shokoohinia, Behzad Zolfaghari, Sedigheh Asgary

**Affiliations:** ^1^ Department of Pharmacognosy, School of Pharmacy and Pharmaceutical Sciences, Isfahan University of Medical Sciences, Isfahan, Iran; ^2^ Ric Scalzo Institute for Botanical Research, Southwest College of Naturopathic Medicine, Tempe, AZ, United States; ^3^ Isfahan Cardiovascular Research Center, Cardiovascular Research Institute, Isfahan University of Medical Sciences, Isfahan, Iran

**Keywords:** cannabis, cbd, HEMP, marijuana, thc

## Abstract

“Hemp” refers to non-intoxicating, low delta-9 tetrahydrocannabinol (Δ9-THC) cultivars of *Cannabis sativa* L. “Marijuana” refers to cultivars with high levels of Δ9-THC, the primary psychoactive cannabinoid found in the plant and a federally controlled substance used for both recreational and therapeutic purposes. Although marijuana and hemp belong to the same genus and species, they differ in terms of chemical and genetic composition, production practices, product uses, and regulatory status. Hemp seed and hemp seed oil have been shown to have valuable nutritional capacity. Cannabidiol (CBD), a non-intoxicating phytocannabinoid with a wide therapeutic index and acceptable side effect profile, has demonstrated high medicinal potential in some conditions. Several countries and states have facilitated the use of THC-dominant medical cannabis for certain conditions, while other countries continue to ban all forms of cannabis regardless of cannabinoid profile or low psychoactive potential. Today, differentiating between hemp and marijuana in the laboratory is no longer a difficult process. Certain thin layer chromatography (TLC) methods can rapidly screen for cannabinoids, and several gas and liquid chromatography techniques have been developed for precise quantification of phytocannabinoids in plant extracts and biological samples. Geographic regulations and testing guidelines for cannabis continue to evolve. As they are improved and clarified, we can better employ the appropriate applications of this uniquely versatile plant from an informed scientific perspective.

## Introduction

*Cannabis sativa* L. is a member of the Cannabaceae family, a small family of annual herbaceous plants that includes the widely cultivated genus, *Humulus* (hops), and eight other genera (Jin et al., 2019). The terms “hemp” and “marijuana” both refer to plants derived from the *Cannabis sativa L.* species, but from different cultivars or chemotypes having either low or high Δ9-THC content, respectively. The general term “cannabis” includes both hemp and marijuana types of plants. (Johnson, 2019). Cannabis has been utilized by several populations for millennia. Central and southeast Asia are considered plausible origins (Steven et al., 2016). It has been claimed that the application of the herb for various purposes dates to 12,000 years ago based on Neolithic evidence found in Taiwan (Li, 1974). Hemp is reputed to be the oldest cultivated fiber plant (Cherney and Small, 2016), and hemp seed and seed oil have historically been used as food (Farinon et al., 2020). Marijuana has long been utilized for recreational as well as medical purposes (Piluzza et al., 2013). The oldest report of the medical use of cannabis dates to 5,000 years ago when it was used for the treatment of fatigue, rheumatism, and malaria (Abel, 2013). The Assyrians, Egyptians, Indians, Persians, Greeks, and Romans are all reported to have used cannabis for medical purposes (Leghissa et al., 2018a).

Classifications for different cannabis species continues to be a highly debated topic amongst taxonomists and botanists (Sawler 2015). Although some experts recognize three different species of Cannabis: *C. sativa*, *C. indica*, and *C. ruderalis* (Pollio, 2016), and others one monospecific species [*C. sativa* L.] with two subspecies [subsp. *sativa* and subsp. *indica* (Lam)] (Small and Cronquist, 1976, UNODC.org), neither of these nomenclature systems accurately reflects the diversity and complexity of modern cannabis plants. Because cannabis species have been cultivated globally over many years to exhibit nearly indistinguishable phenotypes with overlapping genotypes, *Cannabis sativa* L. varieties are now most accurately identified by cultivar or chemotype, including specific cannabinoid profiling and even subtyping (Sarma et al., 2020). Recently proposed suggestions for chemotyping will be discussed.

Although it has valuable nutritional capacity, hemp material was initially cultivated mainly to produce textiles and ropes. Due to expanded applications of synthetic fibers over the past two centuries, its cultivation decreased precipitously. In recent years, hemp regained popularity due to the rediscovery of its nutritional benefits, economic value, and variety of medicinal uses (Farinon et al., 2020) Despite the resurgence of hemp, in ongoing efforts to curtail recreational drug use, several countries and states have restricted the cultivation of cannabis varieties with high concentrations of Δ9-THC, the primary psychoactive metabolite of the plant (Aguilar et al., 2018).

For the past several decades, a lack of understanding and proper differentiation between hemp- and marijuana-type plants has slowed the development of cannabis research on the potential health benefits of the plant (Farinon et al., 2020). And despite having clearer definitions, improper use of these terms is still regularly seen in the literature. It is pertinent to clarify terminology and provide useful tools and legal definitions to differentiate between food, drug, and supplement derivatives of cannabis.

In this review we focus mainly on the question, “What is essential for defining *Cannabis* as a food, supplement, or drug?” We also investigate the nutritional potential of hempseed and seed oil, the medicinal benefits of certain phytocannabinoids (one of the predominant phytochemical groups in Cannabis) as drugs and supplements, and the regulatory status of cannabis and cannabis products across the globe. Finally, we review several analytical techniques for the detection and quantification of cannabinoids in plants extracts, biological samples, and cannabis products.

### Definitions, Production Practices, and Uses

Cannabis terminology has been a significant source of confusion for many. In public opinion at large, marijuana is often viewed as a plant purposed solely for recreational use, which has overshadowed the high value of medical cannabis, hemp-type plants, and hemp products in nutrition, the health and wellness industry, scientific and medical communities, and the global economy (Cadena, 2018). Because of this, it is increasingly important to implement clear nomenclature based on the literature, legal definitions, and the recommendations of agencies specializing in this controversial plant. Here we explore some of the current definitions and nomenclature:

Fundamentally, “marijuana” refers to cultivars of cannabis with psychoactive potential used for both recreational and therapeutic purposes, and “hemp” refers to non-intoxicating cultivars of cannabis with various end uses including fabrics and textiles, nutraceuticals, pharmaceuticals, food products, beverages, oral and topical self-care products, veterinary products, and other manufactured and industrial goods. The term “industrial hemp,” in some cases, may be used interchangeably with “hemp” (Johnson, 2019; Crini et al., 2020).

Officially, based on the U.S. 2018 Farm bill, hemp is now legally defined as follows:

“The plant *Cannabis sativa* L. and any part of that plant, including the seeds thereof and all derivatives, extracts, cannabinoids, isomers, acids, salts, and salts of isomers, whether growing or not, with a Δ9-tetrahydrocannabinol (Δ9-THC) concentration of not more than 0.3 percent on a dry weight basis” ((7 U.S.C. §5940(b)(2)).

Marijuana is still broadly described in the 1970 Controlled Substances Act (CSA) without any limits for Δ9-THC or any other cannabinoid:

“The term marijuana means all parts of the plant *Cannabis sativa* L., whether growing or not; the seeds thereof; the resin extracted from any part of such plant; and every compound, manufacture, salt, derivative, mixture, or preparation of such plant, its seeds or resin.” (21 U.S.C. §802(16)).

This definition of marijuana excludes the mature stalks of *Cannabis sativa* L. and products derived from the stalks (e.g. fiber), as well as sterilized seeds incapable of germination, and any certain preparations of viable seeds, such as oil or cake, provided those preparations do not contain resin.

The institution of the above 2018 Farm bill effectively removed hemp from the legal definition of marijuana originally set forth in the CSA in 1970. The CSA had classified cannabis, including hemp, marijuana, and all associated cannabinoids, as a Schedule I controlled substance. The removal of hemp from controlled substance status allowed for agricultural cultivation of hemp plants on U.S. soil as well as greater federal oversight of hemp in commerce, including commodities like food and supplements. Although the separation of hemp from marijuana allowed significantly more freedom for hemp growers and product manufacturers, as well as researchers, clinicians, and consumers, the change also lead to some new aspects of confusion, specifically surrounding use of the isolated compound, cannabidiol (CBD). We will later explore the nuances of CBD applications in food, pharmaceuticals, and supplements.

The terms hemp and marijuana are both affiliated with lengthy cultural and political histories, and in the case of marijuana, polarizing ones. Today, these terms are no longer fully adequate to describe the numerous varieties and phytochemical complexities found in modern day cannabi*s*, nor accurately describe their potential applications. In 2020, in attempts to improve existing cannabis nomenclature, the United States Pharmacopeia (USP) Cannabis Expert Panel (CEP) suggested, due to cannabis’s complex secondary metabolome and highly variable distribution of chemical constituents, that the following three broad categories be adopted for classifying cannabis based on phytocannabinoid chemotype: 1) tetrahydrocannabinol (THC)-dominant chemotype; 2) intermediate chemotype with both THC and cannabidiol (CBD); and 3) CBD-dominant chemotype (Sarma et al., 2020). They also proposed that these categories could be further subcategorized by other cannabinoids and/or mono- and sesquiterpene profiles as we continue to learn more about the therapeutic potential of these other compounds in humans (Sarma et al., 2020).

Hemp and marijuana plants generally share a common genetic trait pool with some predictable functional genetic variations; however, distinct genetic variations have also been observed amongst cannabis cultivars (particularly hemp cultivars) spanning the entire genome, not just genes related to THC or cannabinoid synthesis (Sawler et al., 2015). Single nucleotide variant analysis demonstrates separation between CBD and THC (Van Bakel et al., 2011). In most cases, the quantitative ratio of phytochemicals tetrahydrocannabinolic acid (THCA) and cannabidiolic acid (CBDA) (THCA:CBDA) may effectively be used for differentiating cannabis cultivars as either CBD- or THC-dominant (or intermediate) as THCA:CBDA ratios strongly reflect underlying genetic nuances in cannabinoid synthase enzymes (THCA and CBDA synthases) and their sequencing, loci distribution, patterns of expression (functional vs. non-functional), and affinities for CBGA (cannabigerolic acid), their shared substrate (Weiblen et al., 2015). Weiblen et al. (2015) also proposed that all three types of cannabis chemotypes (CBD- or THC-dominant, or intermediate) likely contain multiple linked loci with both THCA and CBDA synthase enzymes; however, identifying the presence of a non-functional CBDA synthase may directly indicate THC-dominance in a plant, as this non-functional enzyme has been selected for over many years where marijuana cultivation has thrived. This novel finding, if proven reliable as an identification method, would allow for rapid screening of THC *vs*. CBD-dominant plant material at the genetic level prior to any plant cultivation.

Based on the genetic biosynthetic pathways of CBD and THC, Fernandez et al., 2020 were able to report a systematic genetic prediction of *Cannabis* chemotypes of 62 European agricultural hemp cultivars. They suggested three main chemotype groupings for *Cannabis sativa* L, similar to the chemotypes proposed by the USP CEP: Chemotype I, or THC-*pre*dominant varieties, in which the ratio of CBD/THC is low (0.00–0.05) and characterized by a THC level >0.3% (in the U.S.; dry weight of reproductive parts in the female plant at flowering time), Chemotype III, or CBD-*pre*dominant varieties, in which the ratio of CBD/THC is 15–25 and, in the U.S., contains <0.3% THC concentration (dry weight of reproductive part in female plants at flowering time), and Chemotype II varieties, or THC-intermediate types, which have both THC and CBD in a content ratio (CBD/THC) of about 0.5–3.0 (Fernandez et al., 2020).

These novel nomenclature systems eliminate much of the confusion surrounding hemp and marijuana, and provide a destigmatized, more objective way of discussing Cannabis for certain applications. These classifications are particularly helpful when discussing the female inflorescence material of Cannabis plants which is cultivated for many different chemotypes and cannabinoid concentrations (both THC and CBD-dominant plants) for therapeutic use. For clarity, we will continue to use the three USP categories suggested above (THC-dominant-, CBD-dominant-, and intermediate), where applicable, for the rest of this article.

In addition to the many chemotypes of plant material, there are also differences in production practices applied to cannabis. Based on different applications, hemp is generally cultivated for three different crops: fiber, seed, and CBD-dominant inflorescence. In contrast, THC-dominant plants are grown solely for their inflorescence for both medicinal and recreational use, as the inflorescence contains the most phytocannabinoids (i.e. Δ9-THC) (Johnson, 2019). Generally, all phytocannabinoids in cannabis exist in resin from the secretory glandular trichomes on the flowering tops of female plants. The numbers of these trichomes are fewer in male plants (Hill et al., 2012). For this reason, female flowers are more valuable for phytocannabinoid production, while male plants are more appropriate for fiber production. Cannabis growers cultivating female plants for their phytocannabinoid production (generally CBD-, THC-, or CBD/THC co-dominant chemotypes) usually remove all male plants to prevent pollinating and seed yielding. Conversely, only male plants are grown to produce hemp fiber and seeds. Some hemp farmers producing fiber and seeds try to prevent flowering to encourage taller stalk growth and less branching (Johnson, 2019), whereas those cultivating hemp for CBD, will promote growth of the plant’s phytocannabinoid-rich flowering tops.

Historically, there were significant visual differences between hemp and marijuana plants. Modern cultivated plants appear more phenotypically similar when grown for cannabinoid production, but some distinctions can still be made. Cultivated hemp for fiber and seed usually has the following characteristics: a single tall stalk, few leaves and branches due to high-density cultivation in order to prevent branching, and very tall, up to 4.5 m tall. In contrast, the flowering, phytocannabinoid-rich Cannabis plants (i.e. THC-dominant, CBD-dominant, or intermediate chemotypes) are usually characterized by short, tightly clustered, bushy, low-density cultivation with many leaves and branches (in order to develop buds and flowers), and are only 1.2 m tall (Johnson, 2019).

### Chemical and Genetic Composition

**FIGURE 1:**
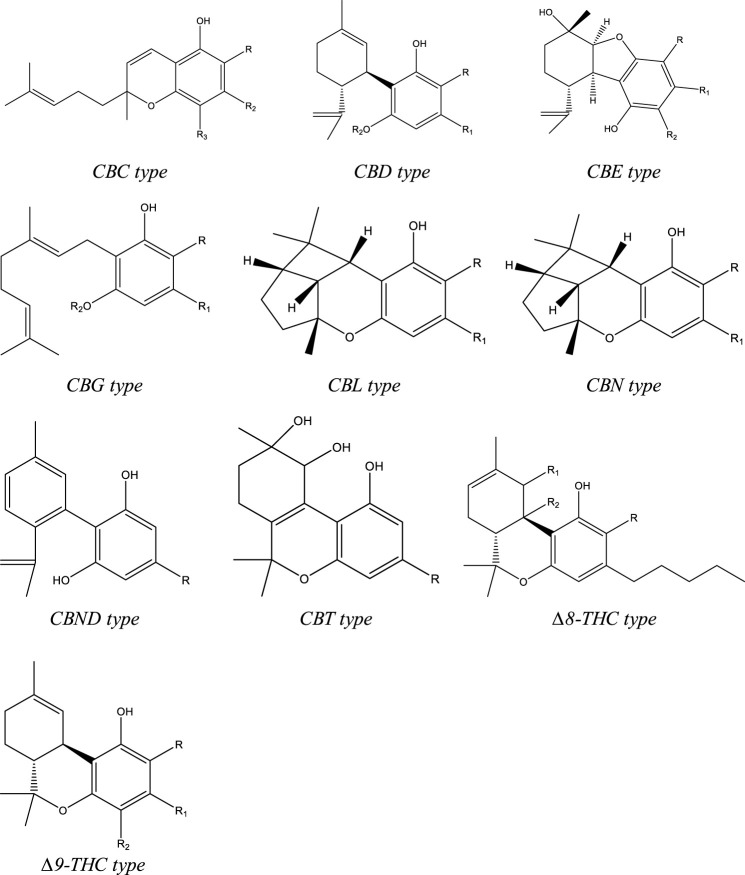
Types of structures of backbones of phytocannabinoids.

To date, more than 500 compounds have been identified from *C. sativa* L., out of which 125 compounds are phytocannabinoids. Structurally these compounds have a C-21 terpenophenolic backbone consisting of an alkyl resorcinol with a monoterpene moiety. Eleven types are described for these cannabinoids based on their structure. They are as follows: cannabigerol (CBG), (−)-∆9-trans-tetrahydrocannabinol (∆9-THC), (−)-∆8-trans-tetrahydrocannabinol (∆8-THC), cannabinol (CBN), cannabidiol (CBD), cannabichromene (CBC), cannabielsoin (CBE), cannabicyclol (CBL), cannabinodiol (CBND), cannabitriol (CBT), and miscellaneous-type cannabinoids (Figure 1). Among the phytocannabinoids, 25 of them are reported to be classified as ∆9-THC types and 10 have been elucidated as CBD types. (Hanus et al., 2016; Radwan et al., 2021). Apart from the cannabinoids, four major categories of non-cannabinoid-type compounds are present, including alkaloids, flavonoids, non-cannabinoid phenols, and terpenes (Radwan 2021).

Although marijuana is technically defined as having greater than just 0.3% ∆9-THC concentration by dry weight in the U.S., today these plants are often bred to have ∆9-THC levels averaging as high as 10–30%. (Crini et al., 2020). Modern hemp plants cultivated for CBD production range anywhere from 5–15% total CBD ((Stack G.M. 2021). The number of cannabis plants cultivated for high CBD:THC ratios has grown exponentially in the past decade. Some researchers are finding that specific CBD-dominant chemovars have a CBDAS gene (CBDA synthase) that synthesizes not only CBDA, the precursor of CBD, but also synthesizes some THCA as a side product (Stack G.M. 2021). With accumulation of CBD, where CBDAS is present, there may be concomitant accumulation of THC that exceeds most legal thresholds prior to the time that the plant accumulates the desired 10% or more total CBD (Stack G.M. 2021). This illustrates the importance of chemovar selection and genetic testing prior to large scale cultivation.

**FIGURE 2:**
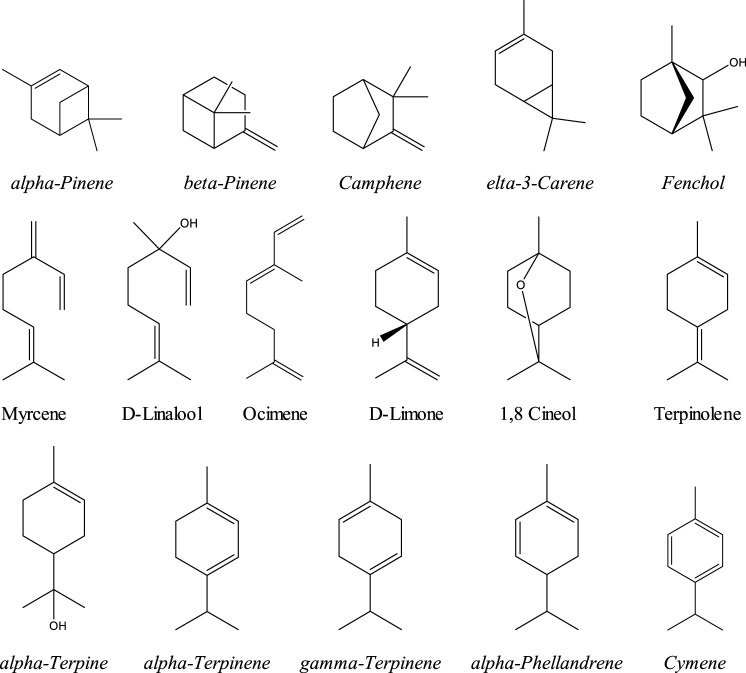
Monoterpenoids commonly encountered in cannabis.

**FIGURE 3:**
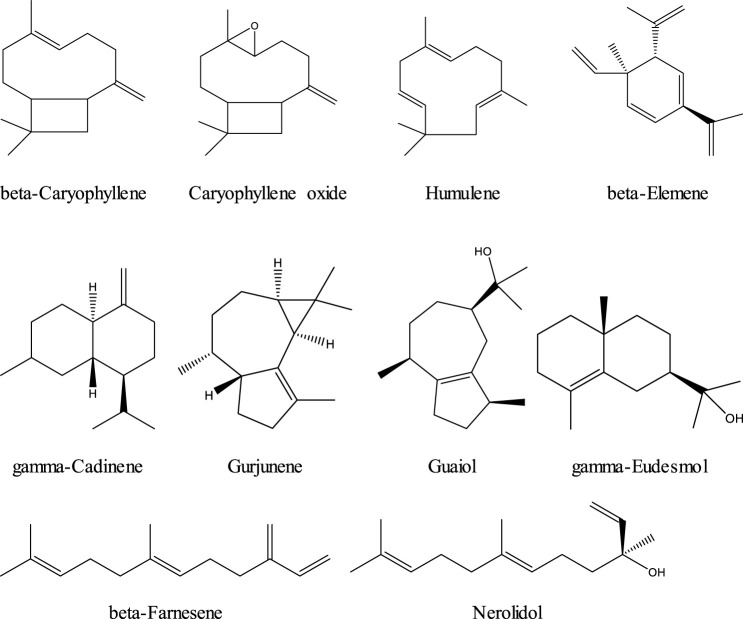
Sesquiterpenoids commonly abundant in cannabis.

Phytocannabinoids are the most abundant constituents in cannabis. Terpenes or isprenoids are the second most abundant constituents, numbering over 200 total terpenes (Russo 2011; Radwan 2021). Typically found in cannabis trichromes at about 10%, and in flowers at about 1%, some chemotypes have been bred to contain as much as 3.5% terpene content or higher in the flowers (Potter, 2009; Fischedick et al., 2010). Although relatively modest in concentration to the cannabinoids, terpenes (mainly mono and sesquiterpenes) are another important class of compounds. Terpenes are of increasing interest to researchers, healthcare practitioners, and the natural products industry as both isolated therapeutic agents and as enhancing compounds in holistic cannabis formulas designed to engage what has recently been labeled “the entourage effect” (Sommano et al., 2020). Variation in terpene content is likely responsible for the more sedative *vs*. stimulating effects of different cannabis cultivars, a notion that had historically been attributed to “indica” *vs*. “sativa” varieties. Terpenes are a family of organic compounds which biosynthetically produced by isoprene units found mainly in plants. Certain cannabis terpenes have been identified and studied in plants such as tea tree, lavender, thyme, basil, *Pinus* sp., frankincense and citrus fruits like lemon and mandarin (Russo & Marcu 2017; Del Prado-Audelo ML et al., 2021). Monoterpenes and sesquiterpenes of cannabis (the volatile compounds) mainly exist in the essential oil which is isolated by hydrodistillation (Radwan et al., 2021). Among terpenes, the monoterpene; *β*-myrcene is mentioned to have the highest prevalence in modern cannabis chemovars in the US (Russo & Marcu 2017). The Sixteen commonly encountered monoterpenes from three classes (acyclic, monocyclic and bicyclic monoterpenes) are shown (Figure 2). The most common terpene that exists in cannabis extracts is the sesquiterpene *β*-caryophyllene. Sesquiterpenes have a 15-carbon skeleton. The most abundant sesquiterpenes of cannabis are drawn (Figure 3) (Russo & Marcu 2017). Other sources cite additional terpenes as most predominant based on samples from more specific geographic locations (Fischedick, 2017; Radwan 2021).

### Pharmacological Effects

Cannabis is known to affect nearly every system in the human body, including but not limited to the central and peripheral nervous systems, as well as the endocrine, immune, gastrointestinal, and musculoskeletal systems. Thousands of articles and over 100 clinical studies have been published on the pharmacodynamics and bioactive effects of cannabis on human psychology, appetite, cognition, sleep, and pain (Russo & Marcu 2017). Only in this century are we are beginning to understand the complexities of these pharmacological actions and interactions, which are largely due to the actions of cannabis on the human endocannabinoid system (ECS) and the “entourage effect” or bioactive synergisms between phytocannabinoids and other compounds within the plant.

The largest body of cannabis research prior to the past two or three decades focused primarily on the ∆9-THC as a medicinal and recreational drug and emphasized its psychoactive attributes and safety profile. In recent years, CBD has garnered more substantial research interest as a non-intoxicating but equally potent therapeutic compound. Structurally, ∆9-THC (THC) was elucidated in 1964 as the main psychoactive compound in cannabis. In 1988, Huestis et al. discovered that THC exerted its psychoactive effects through agonizing cannabinoid receptor 1 (CBR1). CBR1 is a G protein coupled receptor (GPCR) distributed widely throughout the central nervous system (CNS). A few years later, Murano et al. identified a second cannabinoid receptor, CBR2, that also exists in the CNS (in lower concentrations than CB1R), but in greater concentrations in the peripheral nervous system (PNS) and immune system. Two endogenous ligands (endocannabinoids), were later discovered to bind to these cannabinoid receptors - N-arachidonoyl ethanolamide (AEA) and 2-arachidonoyl glycerol (2-AG) (Lader et al., 2009). AEA and 2-AG are physiologic lipid-based retrograde neurotransmitters that interact with the ECS receptors and proteins in a similar manner to exogenous phytocannabinoids CBD and THC.

The pathways by which exogenous cannabinoids such as ∆9-THC and CBD interact with the human endocannabinoid system (ECS) are complex and only partially understood. Definitively, THC has been shown to directly bind CB1 and CB2 receptors, exerting psychoactive and other neurologically-mediated effects. CBD does not directly bind CB1 or CB2 receptors, exhibiting a low affinity for both, but can antagonize synthetic agonists of cannabinoid receptors suggesting a negative allosteric modulatory effect (Freeman et al., 2019; Premoli et al., 2019). Although CBD does not agonize CBR1 or CBR2, it exhibits several cannabinoid receptor-independent activities including direct agonism of TRPV1 and 5-HT1A receptors, enhancement of adenosine receptor signaling, upregulation of peroxisome proliferator activated receptor gamma (PPARγ) protein expression, and suppression of GPR55 receptor activity (Russo & Marcu 2017). CBD is non-intoxicating with a wide therapeutic index and acceptable side effect profile, and has pre-clinically exerted antipsychotic, intestinal anti-prokinetic, neuroprotective, anti-proliferative, anti-ischemic, vasorelaxant, analgesic, anxiolytic, anti-inflammatory, and antiepileptic effects (Noreen et al., 2018; Cerino et al., 2021). CBD also has been shown to negate the unpleasant side effects of THC, including anxiety, psychosis, tachycardia, and drowsiness through negative allosteric modulation of CB1 receptors. (Laprairie et al., 2015).

Other prominent cannabaninoids of pharmaceutical interest include the following: cannabigerol (CBG), cannabichromene (CBC), cannabinol (CBN), tetrahydrocannabivarin (THCV), cannabidivarin (CBDV), and the acidic cannabinoid precursors cannabidiolic acid (CBDA) and tetrahydrocannabinolic acid (THCA). Most of these cannabinoids are still being investigated in preliminary *in vitro* and animal model research studies; however some mechanisms of action have been elucidated and reported in the literature. CBG, for example, has weak affinity for CB1 and CB2 receptors, but is a potent α2-adrenoreceptor agonist, an efficient serotonin 5-HT_1A_ receptor antagonist, and might activate a number of other dominant receptors involved in heat/cold sensitization, pain, and inflammation *via* antagonism of TRPV8 receptors and stimulation of TRPV1, TRPV2, TRPA1, TRPV3, and TRPV4 (Cascio et al., 2010; De Petrocellis & Di Marzo, 2010; De Petrocellis et al., 2011; Morales et al., 2017)). CBC can have profound therapeutic effects on inflammation and pain through CB2 receptor activity and stimulation and desensitization of TRP ankyrin-type 1 (TRPA1) cation channels, interactions with TRPV3 and TRPV4 cation channels, and desensitization of TRPV2 and TRPV4 channels (De Petrocellis et al., 2012; Cascio & Pertwee, 2014). CBC has also been shown to relieve pain in mice by augmenting the analgesic effects of THC when the two are co-administered (Davis & Hatoum, 1983; Maione et al., 2011; Cascio & Pertwee, 2014), and can increase concentrations and prolong the duration of endocannabinoids like anandamide through its interactions with TRP channels (Shinjyo & Di Marzo, 2013; De Petrocellis et al., 2011, 2012). CBN is being investigated for several topical therapeutic targets for skin conditions like psoriasis, burns, and MRSA infections, *via* inhibition of keratinocyte proliferation, TRPV2 agonism, and direct antimicrobial activity, respectively (Wilkinson & Williamson, 2007; Appendio et al., 2008; Qin et al., 2008; Russo, 2014). In addition, CBN is an important marker compound of THC degradation in plant material, an artifact that occurs naturally with storage and may be expedited by high heat exposure as the oxidation byproduct of THC (Upton et al., 2013). THCV, a propyl analogue of THC, exhibits concentration-dependent activity at CB1 receptors and may perform as an agonist or act as an antagonist, accordingly (Pertwee, 2008). THCV demonstrates anticonvulsant attributes in mouse pyriform cortex and cerebellum (Hill et al., 2010), and may promote weight loss and other cardiometabolic benefits (Riedel et al., 2009). CBDV has been reported to have significant anticonvulsant activity and is potentially of equal therapeutic value to CBD in treating epilepsy, especially focal/partial onset seizures (Williams, Jones, & Whalley, 2014). CBDV also activates or blocks a wide range of cation channels, depending on the concentration and experimental conditions, including TRPA1, TRPM8, and TRPV channels 1–4. Of additional importance, CBDV inhibits the cellular uptake of anandamide and inhibits endocannabinoid degradation through modulating enzymes diacylglycerol lipase and N-acylethanolamine-hydrolyzing acid amidase (NAAA) (Pertwee & Cascio, 2014). CBDA, the acidic precursor to CBD, shares the same enhancing activity of CBD at 5-HT_1A_ receptors, even up to ten times higher than the activity of CBD, but does not show agonism or antagonism at CB1 receptors (Bolognini et al., 2013; McPartland et al., 2015). CBDA can also inhibit COX1 and COX2, and, at low concentrations (between 1 and 10 μM), targets GPR55, TRPA1, TRPV1, and TRPM8 (Takeda et al., 2008). THCA, like CBDA, is the acidic precursor of THC, representing up to 90% of total THC in the plant prior to prolonged storage, UV exposure, or heat (Moreno- Sanz, 2016). THCA, like THC, is an agonist of CB1 and CB2 receptors, but there is discrepancy as to which (THC or THCA) has greater affinity (Verhoeckx et al., 2006; Rock et al., 2013; Rosenthaler et al., 2014). Although several cannabinoids have been shown to deposit in brain tissue (Alozie, et al., 1980; Deiana et al., 2012), THCA seems to have limited access to the CNS or ability to cross the blood brain barrier (BBB), likely due to its carboxylic acid group (Moreno-Sanz et al., 2016). This means it also does not elicit any psychoactivity. Preliminary research suggests THCA exhibits antineoplastic, neuroprotective, and anti-inflammatory activities *via* modulation of COX pathways, TRP cation channels, and other immunomodulatory and cell signaling pathways.

In terms of pharmacological effects of cannabis, where there is strong support for the cannabinoids, there is an equal amount of hesitancy in embracing the therapeutic effects of the terpenoids. Although low in concentration in plant material and final cannabis preparations, several aromatic terpenes, especially the monoterpenoids, have been shown to have remarkably potent physiologic effects when administered in humans and animals *via* inhalation in small amounts ((Falk et al., 1990; Falk et al. 1991; Falk et al. 1993). Terpenes are lipophilic, allowing them to penetrate the skin and the blood brain barrier (Buchbauer et al., 1993). The subsequent activity of terpenes in the CNS seems to be part pharmacological and part psychological, where the psychological effects actually predominate when fragrances are inhaled and the sense of smell is stimulated *via* G-protein coupled (odorant) receptors (Buchbauer, 2010). In addition, when scent processing of odor information is prevented, pharmacological effects that might be overridden when fragrances are inhaled become more evident (Buchbauer, 2010). Though most often studied for inducing stimulant or sedative psychoactive effects on the nervous system, terpenes also exhibit marked pharmacological versatility outside of the nervous system. Some additional biological effects are related to their antioxidant, antiviral, antinociceptive, antiphlogistic (anti-inflammatory), and drug penetration enhancing effects (Buchbauer, 2010). These effects are mediated by neuronal and muscle cell ion channels, interactions with cell membranes, second messenger systems and enzymes (Bowles, 2003; Buchbauer, 2010). B-myrcene, one of the most prevalent terpenes found in all nearly all cannabis plants, increases CB1 receptor saturation, thereby enhancing the psychoactive effects of THC (Wedman-St.Louis 2020). Linalool, a terpene also found in mint, lavender, and cinnamon, may increase serotonin and dopamine levels in the brain (Wedman-St.Louis). a-Pinene binds GABA_A_-Benzodiazepine receptors (GABA_A_-BZD) in the brains of rat models, acting as a partial modulator, and may also display acetylcholinesterase inhibitor activity, either alone or in combination with other terpenes commonly found in the same plants (Salehi B, 2019) *β*-Caryophyllene is a potent selective agonist for CB1 receptors which, synergistically, could potentiate the antipruritic and gastro-protective activities of THC and the anti-inflammatory effects of CBD (Gertsch et al., 2008; Russo & Marcu 2017).

Finally, hemp food products can also induce some important pharmacological effects. Phytol, a major diterpene from hemp seed oil has been shown to have anti-cancer as well as antioxidant activities (Vetter et al., 2012). Several studies suggest antihypertensive effects of hydrolyzed hemp seed proteins due to their inhibitory effects on angiotensin-converting enzyme and renin (Aluko et al., 2017). There is also a patented hemp seed flour which was claimed to have an HDL boosting effect and may be beneficial in preventing cardiovascular disease (Guang et al., 2010).

### Food Derivatives

Many food products can be derived from hemp, most of which are made from hemp seed. Common hemp seed food products include bulk or packaged raw hulled hemp seeds, hemp seed oil, hemp protein “concentrates” or powders, and hemp milk, among others. The use of hemp in food products has continued to increase in popularity since the 2018 Federal Farm Bill removed hemp from the Controlled Substances Act. The bill also separated hemp from marijuana and allowed for the interstate transportation of seeds, plants, and processed hemp products. As previously stated, hemp refers to *C. sativa* with less than 0.3% THC by dry weight. For legal and safety reasons, the level of THC must also be determined in final hemp products and included in the label (Ruphasinghe et al., 2020). Oral intake of as low as 2.5 mg THC per day can cause intoxicating effects in adults (Ross et al., 2000). BgVV recommended a maximum limit of 1–2 μg/kg THC per day (BgVV, 1977). Consumption of higher doses in food products should be avoided due to health threatening consequences. To be precautionary, it is important to consider variations in an individual’s sensitivity, drug metabolism, and food-drug interactions. It should also be noted that hemp ingredients from plant parts other than the seeds (including the flower) are not allowed as food ingredients. Non-seed ingredients are prohibited regardless of whether they are added to the food item by the grower/producer, manufacturer, retailer, or by the consumer.

Hemp seeds are a highly nutritious food. Due to the high nutritional value of hemp seed, it could be a complete food source for mankind. Each 100 g hemp seed provides 500–600 Kcal energy (Rodriguez-Leyva et al., 2010). Hemp seed usually contains 25–35% lipids, 20–25% proteins, and 20–30% carbohydrates as well as vitamins (thiamine, riboflavin, pyridoxine, vitamin E and C), minerals (mainly magnesium and iron), flavonoids, tocopherols (alpha, beta, gamma, and delta tocopherols), terpenes, phytosterols and bioactive peptides (Irakli et al., 2019).

A very low but detectable amount of THC is present in hemp seeds, which mainly exists on the seed surface that seems to be a contaminant from glandular trichomes at harvest time. Cultivating and handling processes should be done in such a way that prevents contamination with high levels of THC (Ross et al., 2000). Like THC, CBD is also found in relatively low amounts in the seed oil (Leizer et al., 2000).

Hemp seed oil includes a large percentage of polyunsaturated fatty acids (PUFA) (72–83%) primarily consisting of linoleic (omega-6) and linolenic (omega-3) acids. The ratio of omega-6: omega-3 in the oil is 3:1 which explains its protective potential against cardiovascular disease, osteoporosis, and eczema (Dunford et al., 2015; Ruphasinghe et al., 2020). From a nutritive point of view, the hemp seed oil is much richer than soybean oil (Crini et al., 2020). Human daily nutritional needs of fats can be provided by only three tablespoons of oil (Leizer et al., 2000). Furthermore, hemp seed oil shows a great antioxidant potential. Tocopherol isomers which exist to a large extent in the oil, as well as terpenes and polyphenols (mainly flavonoids), are responsible for this activity (Kriese et al., 2004; Andre et al., 2016; Ruphasinghe et al., 2020). The hemp seed oil also contains a noteworthy amount of phytosterols. It has been noted that plant sterols and stanols exert low-density lipoprotein (LDL) lowering effects in humans (Klingberg et al., 2013).

It is important not to heat the seeds during oil extraction. Unrefined and cold pressed oil is preferred. Cooking the oil produces toxic trans-fatty acids and should be avoided. In addition, unsaturated oil is vulnerable to oxidation, therefore the oil has a very short shelf life and must be protected from air, heat, and light (Crini et al., 2020).

Hemp seed protein is abundant in edestin and albumin, which makes it easily digestible, and provides a beneficial supply of essential amino acids, particularly arginine and glutamic acid (Odani and Odani, 1998; Callaway, 2004). Hemp seed flour is free from gluten and is not known to have any known allergens, hence it does not cause any problem in individuals with celiac disease (Dunford et al., 2015).

Of the many nutritious hemp-derived food products, consumption of hemp seed oil is the most popular. In addition to the more common hemp seed oil, flour, and protein powder, the market of hemp food products continues to expand and now includes a range of novel products from sauces, butter, pasta, and cheese, to energy bars, chocolate, ice cream, and sweets (Klein 2017; Crini et al., 2020).

It should be noted that CBD, the cannabinoid typically found in highest concentration in the inflorescence of hemp plants cultivated for medicinal use, is naturally present, along with other cannabinoids and phytochemicals, in hemp products made from Cannabis inflorescence. There is, however, little to no CBD naturally found in hemp food products like seeds or seed oil, flour, or protein. With rising interest in the beneficial therapeutic effects of CBD and the passage of the 2018 Farm Bill, many retailers began selling CBD-infused foods, beverages, and personal care items, presuming CBD also fell under “hemp” food and supplement regulations; However, CBD was approved as the active ingredient in a seizure medication called Epidiolex in June of 2018. As the active ingredient in a pharmaceutical drug, CBD cannot legally be used as an additive in food or supplements in the U.S. The European Commission, however, has put CBD-containing supplements in the novel foods category (European Commission, 2019). CBD-dominant full-spectrum or broad-spectrum hemp products (<0.3% THC) with naturally occurring cannabinoids, including CBD, are still able to be consumed as food or supplements, but using CBD in any other context is not allowed. CBD and CBD-infused products have remained in the market despite FDA claims of the illegality of CBD supplements and consumers should be aware of these distinctions. Daily intake of 1–2 mg/kg CBD is implausible to be unsafe (Cerino et al., 2021); however, after identifying concerns of hepatotoxicity, this limitation was done to reduce potential life-threatening adverse effects and prevent serious drug or supplement interactions (Cohen et al., 2019).

### Pharmaceutical Drug Derivatives

**FIGURE 4:**
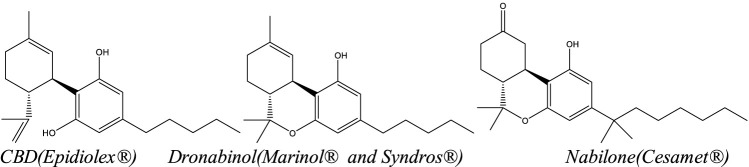
Structures of the active ingredients of FDA-approved cannabinoid drugs.

The United States FDA has approved four Cannabis-related pharmaceutical products to date: one natural, Cannabis-derived drug product—Epidiolex—which contains highly purified CBD, and three synthetic THC-related products: Marinol (dronabinol), Syndros (dronabinol), and Cesamet (nabilone) (Figure 4). Although both drugs containing dronabinol (Marinol and Syndros) are synthetic, dronabinol is also a naturally occurring compound found in Cannabis. In contrast, nabilone is not naturally occurring; it is a man-made drug with structural similarities to THC. In addition to the four cannabis-related products available in the U.S., a product called nabiximols (Sativex), a naturally sourced full spectrum botanical extract with ∼1:1 mixture of THC:CBD in an oromucosal spray, is currently marketed legally in over 25 countries including Mexico, Canada, Australia, the United Kingdom and parts of Europe (Namdar et al., 2020; Pearlson 2020). A Dutch-based company, Bedrocan, also offers five different GMP certified, pharmaceutical grade cannabis products with varying THC:CBD ratios (e.g. THC-dominant, CBD-dominant, and intermediate) with complete terpene profiles. Bedrocan’s products are available in Australia, South Africa, most of Europe, parts of the United Kingdom and Sweden (Pearlson 2020). Finally, a CBD based transdermal gel called Zygel (Zynerba), is being tested in various stages of clinical trials for the treatment of Fragile X Syndrome and other neuropsychiatric conditions including Autism Spectrum Disorder (ASD) and 22q (ClinicalTrials.gov, Urits et al., 2019).

Cannabinoid drugs each fall under a different level of controlled substance regulation (regulations initiated by the CSA and enforced by the DEA), based on each drug’s risk for abuse and physical and/or psychological dependence (Joy et al., 1999). A drug will occasionally change in status with new legislation. Marinol (dronabinol) was originally a schedule II controlled substance, and is now listed under schedule III, indicating it has lower risk for abuse than schedule I or II substances, and has risk of low-to-moderate physical dependency or high psychological dependency if abused (Joy et al., 1999). Syndros (dronabinol) and Cesamet (nabilone) controlled substances are both under schedule II. Epidiolex, a drug containing <0.1% THC, was originally classified in schedule I, but after the hemp farm bill was passed in 2018, it was reduced to schedule V, the least restrictive class of controlled substances (Verma et al., 2021). In April of 2020, Epidiolex was entirely descheduled and is no longer subject to the tracking and monitoring requirements of the CSA. The term “medical marijuana” does not refer to any pharmaceutical drug, but rather use of the whole cannabis plant (with >0.3% THC) as medicine in any product (dried flower, resin, tincture, capsule, etc.) or delivery system (inhalation, oral, sublingual, topical, etc.) (Ebbert 2018). Despite medical and recreational marijuana becoming increasingly legal in several states, medical marijuana is still federally illegal and is classified as a schedule I controlled substance. Physicians may write prescriptions for medical marijuana for patients with certain state-approved medical conditions, which vary widely by state (Ebbert 2018). Hemp, referring to whole plant cannabis with <0.3% THC, was officially removed from the CSA in 2018, so it is no longer a controlled substance and is now regulated under the category of products “generally regarded as safe” (GRAS).

In the U.S., dronabinol (Marinol) was the first FDA approved cannabinoid drug in 1985. By 1999, annual sales of Marinol had reached over $20 billion, and it was primarily being used (>80% of patients) in the treatment of HIV and AIDS as an appetite stimulant (Joy et al., 1999). Marinol is an oil-based capsule taken orally and, since 1992, has been approved to treat two different conditions: chemotherapy-induced nausea and vomiting (CINV) in cancer patients unresponsive to traditional antiemetics and cachexia in AIDS wasting syndrome (Joy et al, l 1999). Preliminary clinical trials suggest dronabinol may also be beneficial for patients with obstructive sleep apnea (OSA) and chronic pain, but further evidence is needed (Fisher 2019). For some patients suffering with chemo-induced nausea and vomiting, oral capsules are an impractical delivery system due to swallowing difficulties, long absorption times (approximately two hours), and high variability in intraindividual absorption and bioavailability (Badowski, 2017; Musty & Rossi 2001). Syndros, a liquid delivery form of dronabinol, was approved in July 2016, as an easier-to-swallow administration option with lower intraindividual variability, faster absorption time (nine minutes *vs*. two hours), and the option to titrate the dosage to clinical effect (Badowski, 2017). Albeit more consistent and tolerable than capsules, because of its liquid delivery system, Syndros may be more easily abused as a drug than Marinol, as it can be converted for inhalation (thus, the schedule II status).

Although many systematic reviews and meta analyses have verified dronabinol’s appetite stimulatory and potent antiemetic effects, both alone and in combination with traditional antiemetics like Odensatron, (Ben Amar, 2006; Mücke 2018; Fisher 2019; Namdar et al., 2020), others suggest dronabinol may be no more effective in treating CINV than modern conventional antiemetics and may also have more side effects (Whiting & Wolff, 2015). In addition, many patients reportedly prefer smoking or vaping naturally derived THC-containing products to using synthetic THC medications (Gieringer 2004; Turgeman 2018; Musty & Rossi 2001; Verma et al., 2021). Some patients also seem to benefit more from CBD and THC combination products (Corroon 2018; Verma et al., 2021) and/or whole plant medical marijuana with its synergistic cannabinoids, terpenes, and flavonoids than using THC alone. It is important to note that FDA approved cannabinoid medications have better dosage regulation and quality control than most natural cannabis products that are state regulated. These approved medications are backed by in depth multi-stage clinical trials evaluating their safety and efficacy, which is not true for most medical marijuana products. Due to historical difficulties in studying cannabis as a schedule I controlled substance, a lot more research is needed in this area (Turgeman 2018; Verma et al., 2021).

The same year that dronabinol was approved (1985), a second oral THC-related drug, nabilone (Cesamet) (Figure 4) was also approved for the treatment of CINV unresponsive to conventional antiemetics and anorexia in AIDS patients (Balter 2017; Taylor 2022). As a synthetic analog of THC (Figure 4), nabilone has enough chemical structural differences from THC that it cannot be converted to THC after ingestion and is not detected as a positive for THC in urinary toxicology screenings (Balter 2017). As a potent cannabinoid receptor agonist, nabilone is reported to have a faster absorption time, stronger potency, increased bioavailability, and longer duration of action compared to dronabinol, thus requiring lower and less frequent dosing (Balter 2017). In addition to its approved uses, early research suggests nabilone may also be useful in treating chronic and neuropathic pain, insomnia, cannabis withdrawal and relapse, posttraumatic stress disorder (PTSD), chronic anxiety, and any combination of these frequently comorbid conditions (Lader., 2009; Balter 2017; Cameron 2014).

In June of 2018, Epidiolex (an oral solution containing 100 mg/ml CBD and <0.1% THC) was approved in the US, initially for seizures associated with two rare and drug-resistant conditions—Lennox-Gastaut Syndrome and Dravet Syndrome—in patients ≥2 ears of age (updated to ≥1 year of age in 2020). In August of 2020, Epidiolex was also approved for use in tuberous sclerosis complex (TSC) related seizures in patients ≥1 year of age (Sekar et al., 2019; Namdar et al., 2020). Cannabidiol (CBD), the primary active constituent in Epidiolex, is metabolized largely through cytochrome P450 (CYP450) enzymes, is a substrate of CYP3A4 and CYP2C19, and also affects the CYP2C family (Abu-Sawwa 2020, Epidiolex [package insert]). Several studies raise concerns regarding drug-drug interactions with CBD and the potential hepatotoxicity of CBD with substantial doses, such as those given with Epidiolex (Abu-Sawwa 2020). Hepatotoxicity has been demonstrated through liver transaminase elevation and increased liver weights of rhesus monkeys and histopathological evidence (Rosenkrantz et al., 1981; Ewing et al., 2019; Huestis et al., 2019; Abu-Sawwa 2020). The application of therapeutic doses in human trials have suggested that hepatotoxicity doesn’t occur in all cases and heavily relies on setting, dose, and timing (Walker et al., 2020). The benefits, in nearly all cases, are determined to outweigh the risks (Abu-Sawwa 2020).

The same manufacturer of Epidiolex—UK-based GW Pharmaceuticals—also manufactures a medicinal cannabis product called Sativex (nabiximols) for the treatment of moderate to severe spasticity in multiple sclerosis patients who have not responded adequately to other MS medications (Russo & Guy 2007; Keating 2017). Unlike any of the above cannabinoid drugs with a single purified or synthesized cannabinoid, Sativex comprises a standardized whole plant *Cannabis* extract with controlled amounts of not just Δ^9^-THC and CBD (2.7 and 2.5 mg per 100 uL single spray, respectively), but also minor cannabinoids, terpenoids, flavonoids, fatty acids, and sterols (Russo and Guy, 2007, GWPharm.com). Patients generally self-titrate the medication, starting with a single spray and increasing up to twelve sprays daily over the course of two weeks. Several systematic reviews of Sativex in uncontrolled MS-related spasticity suggest it has long term benefit in reducing spasticity, especially as adjunctive treatment, and is also generally well tolerated and safe, does not typically result in dependency or abuse, and has low risk for negative psychoactive effects (Russo and Guy 2007; Keating 2017; Rice and Cameron, 2018; Fragoso 2020).

In 2017, the committee on health effects of marijuana from the National Academies of Sciences, Engineering, and Medicine (U.S.) by investigating more than 10.700 abstracts, published an evidence-based review of the health effects of cannabis and cannabinoids. Regarding the efficacy of cannabis and cannabinoids (either natural or synthetic) weight of evidence was categorized mainly as “conclusive evidence”, “substantial evidence”, “moderate evidence”, ”limited evidence” and “no or insufficient evidence” based on the clinical trials (Table 1) (National Academies of Sciences, Engineering, and Medicine, 2017).

**TABLE 1:** Health effects of cannabis and cannabinoids.

Plant Material	Effects of Cannabis and Cannabinoids (Natural or Synthetic)	Effective/In-effective	Weight of Evidence
Cannabis	Treatment of chronic pain in adults	Effective	Conclusive or substantial evidence
Oral cannabinoids	Treatment of chemotherapy-induced nausea and vomiting (antiemetic)	Effective	Conclusive or substantial evidence
Oral cannabinoids	Improving patient-reported multiple sclerosis spasticity symptoms	Effective	Conclusive or substantial evidence
Cannabinoids, primarily nabiximols	Improving short-term sleep outcomes in individuals with sleep disturbance associated with obstructive sleep apnea syndrome, fibromyalgia, chronic pain, and multiple sclerosis	Effective	Moderate evidence
Cannabis and oral cannabinoids	Increasing appetite and decreasing weight loss associated with HIV/AIDS	Effective	Limited evidence
Oral cannabinoids	Improving clinician-measured multiple sclerosis spasticity symptoms	Effective	Limited evidence
Cannabis	Improving symptoms of Tourette syndrome	Effective	Limited evidence
Cannabidiol	Improving anxiety symptoms, as evaluated by a public speaking test, in individuals with social anxiety disorders	Effective	Limited evidence
Nabilone	Improving symptoms of posttraumatic stress disorder	Effective	Limited evidence
Cannabis	Better outcomes (i.e., mortality, disability) after a traumatic brain injury or intracranial hemorrhage	Effective	Limited evidence
Cannabinoids	Improving symptoms associated with dementia	In-effective	Limited evidence
Cannabinoids	Improving intraocular pressure associated with glaucoma	In-effective	Limited evidence
Nabiximols, dronabinol, and nabilone	Reducing depressive symptoms in individuals with chronic pain or multiple sclerosis	In-effective	Limited evidence
Cannabinoids	Cancers, including glioma	Effective	No or insufficient evidence
Cannabinoids	Cancer-associated anorexia cachexia syndrome and anorexia nervosa	Effective	No or insufficient evidence
Dronabinol	Symptoms of irritable bowel syndrome	Effective	No or insufficient evidence
Cannabinoids	Epilepsy	Effective	No or insufficient evidence
Cannabinoids	Spasticity in patients with paralysis due to spinal cord injury	Effective	No or insufficient evidence
Cannabinoids	Symptoms associated with amyotrophic lateral sclerosis	Effective	No or insufficient evidence
Oral cannabinoids	Chorea and certain neuropsychiatric symptoms associated with Huntington’s disease	Effective	No or insufficient evidence
Cannabinoids	Motor system symptoms associated with Parkinson’s disease or the levodopa-induced dyskinesia	Effective	No or insufficient evidence
Nabilone and dronabinol	Dystonia	Effective	No or insufficient evidence
Cannabinoids	Achieving abstinence in the use of addictive substances	Effective	No or insufficient evidence
Cannabidiol	Mental health outcomes in individuals with schizophrenia or schizophreniform psychosis	Effective	No or insufficient evidence

### Regulatory Status

Before the late 1950s, the production of hemp was encouraged by the U.S Department of Agriculture (USDA) (Johnson, 2019). After introducing THC as the psychotropic constituent of *Cannabis sativa* L., authorities started to make policies against the expansion of cultivating all types of the cannabis plant without distinguishing between low and high THC varieties (Farinon et al., 2020). Cannabis was internationally banned by the Single Convention on Narcotic Drugs in 1961 and classified in schedule I, along with substances like heroin. Schedule I controlled substances are concluded to be highly addictive, have great potential for abuse, and/or have capacity for use as precursors for other illegal drugs. In addition, the Convention on Psychotropic Substances in 1971, listed THC in schedule I due to its potential for abuse, threat to public health, and low therapeutic potential. In 1988, the United Nations Convention against Illicit Traffic in Narcotic Drugs and Psychotropic Substances prohibited “the production, manufacture, supply, distribution, sale, transportation, import or export of any narcotic drug or psychotropic substance.” It also prohibited possession of these substances and cultivation of plants under the legislation of states parties (UNODC, 2013). As a result, the importance of scientific and medical research regarding cannabis was marginalized (Aguilar et al., 2018).

Today, the regulatory aspect of cannabis that is of greatest concern in most jurisdictions is the employment of strict THC limits to determine which plants fall under “hemp” status and can be legally cultivated, sold, and marketed as such. A limit of 0.3% THC (dry weight basis) in “young, vigorous leaves of relatively mature plants” was a guideline arbitrarily adopted by Small et al. (1976) for this purpose, and many countries, including the U.S., have established this same criterion of a 0.3% THC limit. The range for THC limits across the globe currently spans between 0.2 and 1.0%, but most countries have chosen 0.2% as the upper limit. Relatively few jurisdictions, including the Swiss government, have increased the legal limit for hemp cultivars to as high as 1.0% THC, a much higher percent than the rest of the European Union (EU), which currently limits THC content to just 0.2%. The European Union (EU) legalized the cultivation of *C. sativa* L. plants for industrial applications in 2013 limiting trade to varieties with less than 0.2% THC dry weight of leaves and flowering parts (EMCDDA, 2018). The EU had initially set the upper limit for industrial hemp to 0.5% THC in 1984, then reduced it to 0.3% in 1987, and finally to 0.2% in 1999. In Canada, cultivating hemp varieties with THC content below 0.3% dry weight of leaves and flowering parts was allowed in 1998. Today, Canada is the largest producer and exporter of hemp products, in particular, hemp-based food and ingredients, around the world (Johnson, 2018). The hemp-producing market of EU is the second only to Canada. Countries including France, Netherlands, Lithuania, and Romania are among the largest producers in the EU (Johnson, 2018). The EU also certifies specific hemp varieties of seeds in the “Common Catalogue of Varieties of Agriculatural Plant Species,” and grants subsidies to farmers using these catalogued seeds as long as they remain <0.2% THC (Glivar et al., 2020).

In each of the above cases, measurements of THC concentrations are usually required to include not just THC, but THC’s carboxylated precursor, THCA, and sometimes its non-enzymatic oxidation byproduct, CBN (Stack G.M. 2021, Glivar T, et al., 2020, Sarma 2020). THCA is more prevalent than THC in fresh cannabis plant material, as well as in samples dried at low temperature that have not been in storage for long (Glivar T, et al., 2020). When THCA is naturally decarboxylated to THC, there is a loss of molecular mass, so calculations for %w/w [%mg of cannabinoid/100 mg herbal drug] for total THC concentration should include the following ratio of 0.877% THCA:

% of Total Δ9 -THC = (% Δ9-THC-A x 0.877) + % Δ9-THC + % CBN.

Similarly, when calculating total CBD concentration, the following should be performed to account for the decarboxylation reaction of CBDA to CBD:

% of Total CBD = (% CBD-A x 0.877) + % CBD.

The preceding paragraphs specifically discuss hemp regulations, which likely define the most important aspect of Cannabis governance today; however, this unique plant presents other regulatory challenges, such as the legalization of medical and/or recreational THC-dominant cannabis, determining which conditions are eligible for medical THC use, discrepancies between federal and state cannabis regulations, and confusion around the use of isolated cannabinoids, like CBD, which are present in full- and broad-spectrum hemp extracts.

In terms of recreational cannabis legalization, Latin America has been the world’s pioneer in facilitating regulations for recreational THC-dominant cannabis. Uruguay was the first country in the world which legalized production, distribution, sale, and consumption of marijuana and its derivatives for purposes other than medical and scientific uses (MacKay & Philips, 2016). Cannabis markets in Uruguay were allowed to sell marijuana for medicinal, scientific, industrial, and recreational purposes under several considerations (Aguilar et al., 2018). After regulatory changes in 2017, Germany was one of the first countries to pronounce that both public health services and private medical insurances should cover medical cannabis treatments (Aguilar et al., 2018). Select countries like Portugal have decriminalized (“eliminating criminal penalties for the unauthorized consumption and possession of small amounts for personal use only of a controlled substance”) the use of marijuana. In Spain, possession of marijuana was decriminalized too, but not its sale. In the Netherlands, use of marijuana has been *de facto* decriminalized (“not applying statutes that penalize the production, distribution, or consumption of a substance to the fullest extent”) (MacKay & Philips, 2016). Also in the Netherlands, Cannabis manufacturer, Bedrocan, has expanded to procure vast ownership over medicinal cannabis production in the country and produces, standardizes and exports large amounts of cannabis products with different THC and CBD concentrations. More strict policies still apply in parts of Asia, as medical cannabis has remained prohibited in Iran, Japan, Vietnam, and Pakistan; but in India, several legal provisions for use of medical cannabis have been permitted (Aguilar et al., 2018; Ghiabi et al., 2018).

In the U.S. although marijuana remains a schedule I controlled substance under federal law which bans the manufacture, distribution, dispensation, and possession of marijuana, several individual states have elected to legalize marijuana for recreational use apart from federal guidelines (Johnson, 2018). In the U.S., marketing of all cannabis-derived products and CBD was banned until a few years ago based on their schedule I status and concerns about their side effects. The 2018 Farm Bill modified the definition of hemp so that hemp was no longer a controlled substance. Hemp cultivation in the US is now under USDA-approved license while growing marijuana is generally prohibited because it is considered to have drug-abuse potential (Johnson, 2019). Regardless of federal restrictions, select states allow for the legal cultivation of cannabis plants both for medicinal and recreational purposes. Dietary supplements of CBD remain federally illegal, but FDA has primarily taken action only against marketers making disease treatment claims. After approving Epidiolex, which contains the active ingredient, CBD, for certain epilepsy conditions, CBD was inevitably assigned pharmaceutical drug status and could no longer be treated like a dietary supplement (Walker et al., 2020).

### Analytical Methods

Based on International Narcotic Control Board (INCB) reports, total globally production of cannabis increased from 1.3 tons in 2000 to 100.2 tons in 2015 (INCB, 2016). Third party cannabis testing laboratories are responsible for analyzing and quality control of this massive amount of raw material as well as any final product containing cannabis, whether THC-dominant, CBD-dominant, or intermediate. In addition to phytocannabinoid potency testing, which is typically done *via* HPLC (high performance liquid chromatography), many cannabis labs also now commonly test for terpene content (typically done *via* GCMS: gas chromatography with mass spectrometry), as well as several contaminants—pesticides, heavy metals (cannabis is a hyperaccumulator), mycotoxins, and residual solvents on derivatives and edibles. For any final cannabis products, in addition to the above analyses, many labs also test for water activity, moisture content, and homogeneity to determine potential for microbial growth and an equal distribution of cannabinoids within the product, respectively. Testing requirements are overseen by specific regulating bodies where cannabis is being grown, traded, or consumed. Labs regularly consult with growers, insurers, producers, distributors, and retailers to ensure final products are labeled and marketed accurately and are safe for consumers. In addition to the above tests, some other tests labs occasionally perform include product stability, flavonoid and lipid concentrations, gender testing, nutrient testing, and leachability studies. The breadth of all things cannabis lab testing is beyond the scope of this article. Our focus will be on phytocannbinoid profiling to determine CBD-*vs.* THC-dominant chemovars and pros and cons of the most common analysis platforms.

Though different analytical methods and guidelines exist, differentiating between CBD- and THC-dominant cannabis chemotypes is no longer a difficult process, due to increased knowledge of biochemical and biomolecular characteristics of *C. sativa* L. and the development of instruments and analytical methods that facilitate detection and exact quantification of cannabinoid content (Farinon et al., 2020; Stefkov 2022). The United Nations suggested identifying cannabinoids fast and easily with color tests—e.g. the Fast Corinth V salt test, the Fast Blue B salt test, and the Rapid Duquenois test. Thin layer chromatography (TLC) in both normal and reverse phase systems, besides visualizing with Fast Blue B salt as a reagent, may be used to detect cannabinoids generally. TLC is typically used for fast screening of cannabinoids and does not provide further information; in addition it has low specificity and sensitivity and false-positive results may be seen (UNODC, 2009; Leghissa et al., 2018a; Citti et al., 2018). An accurate and reproducible rapid high-performance TLC (HPTLC) method was developed for quantitative analysis of total THC quantification (decarboxylated THCA + THC + CBN) along with a qualitative fingerprint of the other primary neutral cannabinoids (CBD, THCV, CBG and CBC), with results comparable to that of an HPLC fingerprint (Fischedick et al., 2009; Stefkov 2022). HPTLC may be helpful in screening for high THC content in hemp and hemp products.

Gas chromatography (GC) is one of the oldest and most common methods used for quantifying cannabinoids. The method is accomplished in a short period (under 20 min) at 300°C with low polarity stationary phases (e.g. 5% diphenyl and 95% dimethyl polysiloxan). Cannabinoids are partly present in the plant in their acidic forms. Quantification of acidic cannabinoids with GC is impossible because they undergo decarboxylation during passage through the hot injection port; hence it is necessary for cannabinoids to be derivatized prior to GC. Derivatizing also results in better peak shape and more volatility (Dussy et al., 2005; Leghissa et al., 2018a). FID and MS commonly are used as detectors for the recognition and quantification of cannabinoids coupled with GC. GC-MS is the most researched and reviewed platform for analytical cannabinoid profiling to date (Citti 2018; Kowalska et al, 2020; Kenezvic et al, 2021). GC-FID provides a more accurate and cheaper quantification than GC-MS because GC-MS requires expensive and not always available equivalent deuterated standards (Citti et al., 2018). Due to its higher efficacy, helium gas generally is preferred over nitrogen and hydrogen in studies as a carrier gas in GC-MS (Lazarjani et al., 2020). Several other GC methods have been reported. Some of these include, GC technology and starch gel electrophoresis (Hillig & Mahlberg, 2004), multiple reaction monitoring (MRM) analysis GC coupled with triple quadrupole mass spectroscopy (Leghissa et al., 2018b), vacuum ultraviolet spectroscopy (GC-VUV) (Leghissa et al., 2018c) and two-dimensional gas chromatography (Omar et al., 2014).

High-performance liquid chromatography (HPLC) is gaining popularity as the botanical chemical fingerprint method of choice, including for phytocannabinoid profiling (Stefkov et al., 2022). The most commonly analyzed cannabinoids include ∆9 -THC, THCA, CBD, CBDA, CBN, CBG, and CBC. Using C18 columns for the stationary phase allows for high resolution and separation of cannabinoids. The addition of 0.1% formic acid to methanol or water for the mobile phase improves peak shapes and enhances resolution (Leghissa et al., 2018a; Citti et al., 2018). It is possible to quantify both neutral and acidic cannabinoids with HPLC (i.e. THC and THCA), due to low-temperature operation. In contrast, HPLC is not appropriate for the analysis of volatile compounds such as terpenes, which are better analyzed *via* GCMS (Lazarjani et al., 2020). Mass spectroscopy (MS) and ultraviolet (UV) are commonly coupled with HPLC as a detector (Lazarjani et al., 2020). UV is cheaper and more straightforward than MS (Leghissa et al., 2018a). Neutral cannabinoids show a UV absorption peak around 220 nm and acidic cannabinoids peak in the range of 270–310 nm (Hazekamp et al., 2005; Citti et al., 2016). MS may provide higher specificity than UV, which makes it possible to analyze complex matrices of cannabis (Citti et al., 2018). To yield further information and improve specificity, the diode array detector (DAD) has been suggested by some authors (Leghissa et al., 2018a). HPLC-DAD differentiates between *Cannabis sativa* L. chemotypes based on different absorption spectra (Peschel & Polit, 2015). In order to differentiate between CBD and CBG, using HPLC-MS is preferred over HPLC-UV because UV does not provide enough resolution in this case, with certainty, in above 10% concentration of extract (Giese et al., 2015; Lazarjani et al., 2020). Some other methods have also been shown to have different advantages in the quantification of cannabinoids including LC-MS/MS and APCI (Grauwiler et al., 2007), HPLC-MS/MS (Aizpurua-Olaizola et al., 2014), and UPLC-qTOF (Lazarjani et al., 2020).

Many labs prefer either GC- or LC- based methods for cannabinoid testing. According to a recent comprehensive literature review of the past four decades of analytical methods reported for cannabinoid profiling in over 220 scientific papers, Stefkov et al., 2022 states that GC- and LC-based methods, at this point in time, exhibit comparable accuracy, selectivity, linearity, sensitivity and precision. Both are used routinely and in investigational analysis of cannabis and cannabis products. HPTLC may soon prove to be superior to either one of these (GC techniques or HPLC) due to the possibilities of lower running costs, quicker analysis run time, automation of sample application and spotting, and ability to analyze multiple samples simultaneously.

In addition to developing a highly sensitive, precise, and affordable instrumental analysis, identification of plant materials, sample selection, and preparation should be taken into account.

Macroscopic and microscopic features are primarily used to identify the plant material. Differential identification is accomplished to figure out the presence of closely related species or other adulterations. HPTLC fingerprint could complete the macroscopic and microscopic techniques, as it provides important data about the major cannabinoids. The USP general chapters <563>, <203>, and <1064> describe general procedures which could be useful in the identification of botanicals (Sarma et al., 2020). A proper sample of plant material should represent the characteristics of the whole batch to make sure of reproducibility in the results of quantification analyses. A non-homogenized or stratified batch may lead to under- or overestimation of cannabinoid content (due to higher levels of cannabinoids in glandular hair trichomes) (Sarma et al., 2020). The USP general chapter <561> explains a valid sampling procedure for botanical drugs. The procedure is based on the weight of the holding container. Contents should be mixed for less than 1 kg containers. For 1–5 kg containers, sampling should be performed by withdrawing an equal amount of plant material from the upper, middle, and lower parts of the container. For above 5 kg containers, three samples must be taken from each of the upper, middle and lower parts (USP <561>, 2019).

Sample preparation varies and depends mainly on the aim of analysis and sample form (solid, liquid, or gas) (Chen et al., 2021). For non-biological samples which range from raw materials and cannabis extracts to semi- (mixture of cannabinoids) or fully-purified (single cannabinoid) materials and formulated cannabis preparations (e.g, edible oil or capsule), analysis of cannabinoids is done to determine the chemical profile of cannabinoids. Cannabis extraction is the process of isolating cannabinoids or terpenes from cannabis trichomes with the purpose of producing medical or recreational supplements and is necessary before any quantification or purification. This process involves several consecutive procedures as follows: Harvesting, trimming the flowers, drying in the absence of sunlight to avoid chemical degradation, milling to decrease particle size, immersion in a solvent (constituents release into the solvent), winterizing (a deep-freezing procedure with the aim of the sedimentation of a viscous black residue called wax), filtration to remove wax from the extract and finally, removal of the solvent using a rotary evaporator. Further purification methods, might be followed according to the final goal, such as crystallization or chromatography. Polar solvents are routinely used for the solvent extraction of semi-polar cannabinoids or terpenes from the flower trichomes e.g, ethanol, methanol, isopropanol, dimethyl ether (Valizadehderakhshan et al., 2021).

Biological specimens (blood, plasma, serum, urine, oral fluid, breath, and hair) typically are analyzed for research purposes and forensic toxicological and drug abuse investigations (Karschner et al., 2020). Several solvent-based extraction approaches are used for solid samples to extract the marker analytes (cannabinoids in this case) and remove any byproducts. Liquid-phase extraction (LPE), solid-phase extraction (SPE), and solvent extraction assisted with microwave, ultrasonic, or pressure methods have been proposed for solid sample preparation. Solid-phase microextraction (SPME) is widely employed for hair samples due to increased automation and potential for high throughput. Liquid samples are mostly involved in the detection of drug abuse cases. Similar to solid samples, liquid samples must be processed for isolation of cannabinoids and elimination of interferences. Liquid-Liquid extraction (LLE) and solid-phase extraction (SPE), in addition to recent microextraction methods including solid-phase microextraction, packed sorbent microextraction, dispersive solid-phase extraction, dispersive liquid-liquid microextraction, and hollow fiber-based liquid-phase microextraction, are used for this purpose. As far as gas samples like a sample of air is typically prepared through capillary microextraction. For aerosols, liquid-phase extraction is utilized after collecting airborne particles (Chen et al., 2021).

## Discussion

Confusion and inappropriate use of the terms “marijuana” and “hemp” is a great limitation in regards to broader acceptance and widespread use of the cannabis plant. Distinctions between hemp and marijuana extend beyond definitions and concentrations of THC, as they differ in chemical and genetic composition, production practices, and especially in their use as medicine, food, and in industrial applications. Hundreds of preclinical and clinical studies have been done to evaluate the efficacy and safety of phytocannabinoids like CBD in different conditions, but the body of evidence from high-quality clinical studies still lacks robust conclusions about CBD’s safety and efficacy in treating diseases other than epilepsy.

Due to the complex nature of the endocannabinoid system (ECS) and its role on several physiologic functions in the body such as mood, memory, inflammation, and appetite, there are still many uncertainties about the way exogenous phytocannabinoids interact with the human ECS, and many areas that still need further research.

In addition, there is an ongoing need for legislative and regulatory intervention in some countries in order to provide a platform for developing research about cannabis and related industries. Making policies that promote the advantages of cannabis and prevent its misuse is necessary, and policymakers should attempt to increase public knowledge about cannabis safety based on current research. Most of the primary issues with cannabis could likely be resolved through identifying and quantifying intoxicating compounds in both original plant material and cannabis products, agreement on universal nomenclature, reporting and labeling guidelines, and continued education for all parties involved—farmers, insurers, policy makers, scientists, physicians, and consumers. Instrumental analytical methods and genetic testing will play a crucial role in achieving this goal. .

Despite all of the recent advances, several cannabis topics remain to be addressed. For example, exploring advantages of natural plant extracts over synthetic drugs, clarifying legal use of full spectrum hemp products *vs*. low dose CBD-containing products *vs*. high dose, purified CBD in prescription medication, and exploring the many benefits of lesser known terpenes and minor cannabinoids. Nevertheless, due to expanding knowledge about cannabis’s value in medicine and industry, and recent changes in legislation allowing for easier access to this plant, novel research findings are continuing to grow and spread around the world.
